# When plans change: Surgical implantation of a transcatheter pulmonary valve in hypoplastic left heart syndrome

**DOI:** 10.1016/j.xjtc.2026.102198

**Published:** 2026-01-09

**Authors:** Sebastián Quiñones-Carrasquillo, Thomas M. Giammarino, Mohammad Alaeddine, Eiri Kisamori, Daniel A. Velez

**Affiliations:** a Department of Cardiac Surgery, Phoenix Children's Hospital, Phoenix, Ariz; b Department of General Surgery, University of Puerto Rico, San Juan, Puerto Rico

**Figure:**
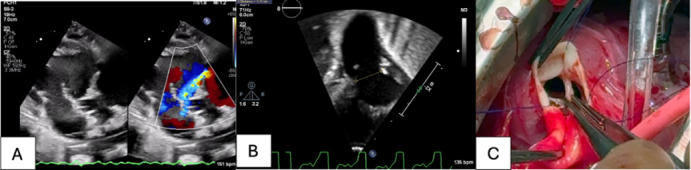
Left pulmonic cusp free margin avulsion with lower forceps holding valve edge.

**Central Message**

This case highlights the technical feasibility of surgical implant of a transcatheter valve in the pulmonary position as a bridge to decision in critically ill neonates with HLHS.

Valve dysfunction in single-ventricle patients can be detrimental at any stage of palliation, leading to increased morbidity and mortality.^1^ Addressing valve dysfunction in neonates may be hindered by severe dysfunction, futile attempts at repair, inadequate patient size for available valve types, or the need to consider bailout strategies such as extracorporeal membrane oxygenation or a ventricular assist device. The Melody valve (Medtronic, Inc) has been used as an alternative in patients with a small right ventricular outflow tract. It has been successfully implanted using the perventricular approach in children weighing as little as 4.7 kg.^2^ However, there are currently no previously described techniques of pulmonary valve replacement in a single ventricle before first-stage palliation. We present the case of a 10-day-old, 3.8-kg, term neonate diagnosed with hypoplastic left heart syndrome (HLHS) with mitral stenosis and aortic atresia who underwent urgent pulmonary valve replacement with a 22-mm Melody valve ballooned to 14 mm. Written informed consent was obtained from the patient's parents, including consent for publication, and Institutional Review Board approval was waived.

## Clinical Summary

**Figure 1:**
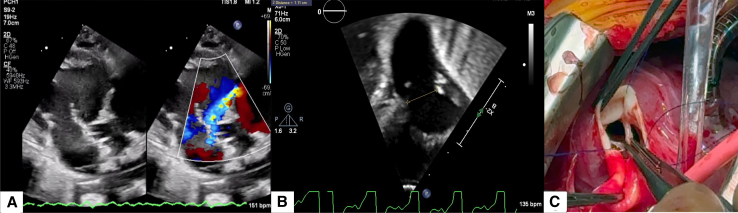
A, Preoperative echochardiography showing new-onset regurgitation with eccentric jet (Video 2). B, Preoperative echocardiography showing pulmonary annulus measuring 11 mm. C, Intraoperative finding of left pulmonic cusp free margin avulsion with lower forceps holding valve edge (Video 3).

**Figure 2:**
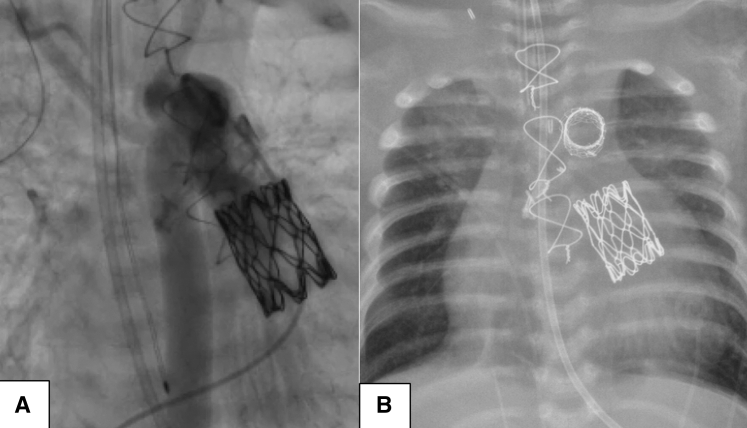
A, Postoperative cardiac catheterization showing well-seated 22-mm Melody valve in the pulmonary position with no insufficiency. B, Chest x-ray showing patent ductus arteriosus stent and 22-mm Melody valve.

A term neonate was transferred to our institution on day of life 0 with worsening hypotension, on mechanical ventilation, with metabolic acidosis, acute kidney injury, increasing inotropic support, and a peripheral oxygen saturation greater than 90%, consistent with early pulmonary overcirculation. The decision was made to control overcirculation using pulmonary blood flow restrictors as a temporizing strategy toward the Norwood procedure. After the flow restrictor implant, echocardiography demonstrated new onset of pulmonary regurgitation (Figure 1, *A*). It was described as a 3-mm defect in the posterior versus the left leaflet of the pulmonary valve. Function was described as moderately decreased. The patient experienced metabolic deterioration and was subsequently placed on venoarterial extracorporeal membrane oxygenation (VA-ECMO). After multidisciplinary discussion, it was decided to attempt repair of the pulmonary valve (Video 1). Surgery was performed via median sternotomy with circumferential dissection of the pulmonary arteries for right and left pulmonary band placement. We transitioned to cardiopulmonary bypass. Immediate control of the ductus arteriosus followed. The heart was arrested with cold crystalloid cardioplegia. The main pulmonary artery was divided, and the pulmonary blood flow restrictors were removed. Inspection of the pulmonary valve revealed an unrepairable left pulmonic free margin avulsion (Figure 1, *C*, Video 2). At this point, the decision was made to replace the pulmonary valve with a Melody transcatheter valve. A 22-mm Melody valve was shortened by folding the metallic triangular edges outward both proximally and distally. The valve was crimped by hand over a 14-mm balloon and positioned across the pulmonary annulus, taking care to leave enough pulmonary artery tissue to achieve closure. On the proximal end, we took the postdilation length of the modified valve (20 mm) into consideration to avoid obstruction of the right ventricular outflow tract or tricuspid valve dysfunction. The radial tension at 14 mm was sufficient to anchor the valve across the annulus (Figure 1, *B*, Video 3). It was also secured with 3 interrupted sutures at the distal end. We were careful to avoid overdilation to maximum circumference to prevent compression of the aortic root or coronary circulation. The arteriotomy was closed with running suture, and bilateral branch pulmonary arteries were banded over a 2-mm dilator. Cardiopulmonary bypass time was 78 minutes with an aortic crossclamp time of 50 minutes. The patient was successfully decannulated from VA-ECMO and supported with low-dose inotropes. Postoperative echocardiography revealed mild decreased right ventricular function with the Melody valve without evidence of stenosis, regurgitation, paravalvular leaks, or outflow tract obstruction. Eventually, the patient underwent successful patent ductus arteriosus stent placement as palliation. Cardiac catheterization revealed a well-seated valve with no stenosis or regurgitation (Figure 2, *A*). The patient continues to demonstrate adequate growth and no change in ventricular or valve function while awaiting further decision in management toward discharge.

## Discussion

This case describes what we believe to be the first successful urgent replacement of the native pulmonary valve using a surgically placed Melody valve in a patient with HLHS and pulmonary regurgitation. The rationale for using the Melody valve included the small size of the child, the complete avulsion of the pulmonic cusp, and the friability of the tissue, which rendered any attempt at suture repair futile. Current data regarding valve replacement in single-ventricle patients, including outcomes, timing, and surgical strategies other than repair, remain limited.^3^ Alshami and colleagues,^3^ using data from the Pediatric Cardiac Consortium, found only 12 of 3807 patients with single-ventricle physiology had undergone semilunar valve surgical or transcatheter replacement. None of these were described before stage I palliation or performed via an open surgical approach. Furthermore, prior reports have described Melody valve use in single-ventricle patients primarily in other positions months after completion of staged palliation, often as a bridge to transplantation or further surgery.^4^^,^^5^ In contrast, our patient underwent surgical Melody valve implantation in the pulmonary position before stage I palliation.

## Conclusions

This case highlights the technical feasibility of surgical implantation of a transcatheter valve in the pulmonary position as a bridge to decision in critically ill neonates with HLHS. Additionally, the patient was successfully decannulated from VA-ECMO and has shown adequate growth since the procedure, with preserved ventricular function and satisfactory valve performance, supporting the short-term functional success of this intervention.

## Conflict of Interest Statement

The authors reported no conflicts of interest.

The *Journal* policy requires editors and reviewers to disclose conflicts of interest and to decline handling or reviewing manuscripts for which they may have a conflict of interest. The editors and reviewers of this article have no conflicts of interest.

## Supplementary Data

Video 1 Preoperative echocardiogram. Video available at: https://www.jtcvs.org/article/S2666-2507(26)00005-2/fulltext.

Video 2 Pulmonic valve cusp avulsion. Video available at: https://www.jtcvs.org/article/S2666-2507(26)00005-2/fulltext.

Video 3 Implantation technique of transcatheter valve into affected valve. Video available at: https://www.jtcvs.org/article/S2666-2507(26)00005-2/fulltext.
