# Supplementary figures and images for: When plans change: Surgical implantation of a transcatheter pulmonary valve in hypoplastic left heart syndrome
Source: JTCVS Tech. 2026 Jan 9;36:102198. doi: 10.1016/j.xjtc.2026.102198 (PMC13069556; doi:10.1016/j.xjtc.2026.102198)

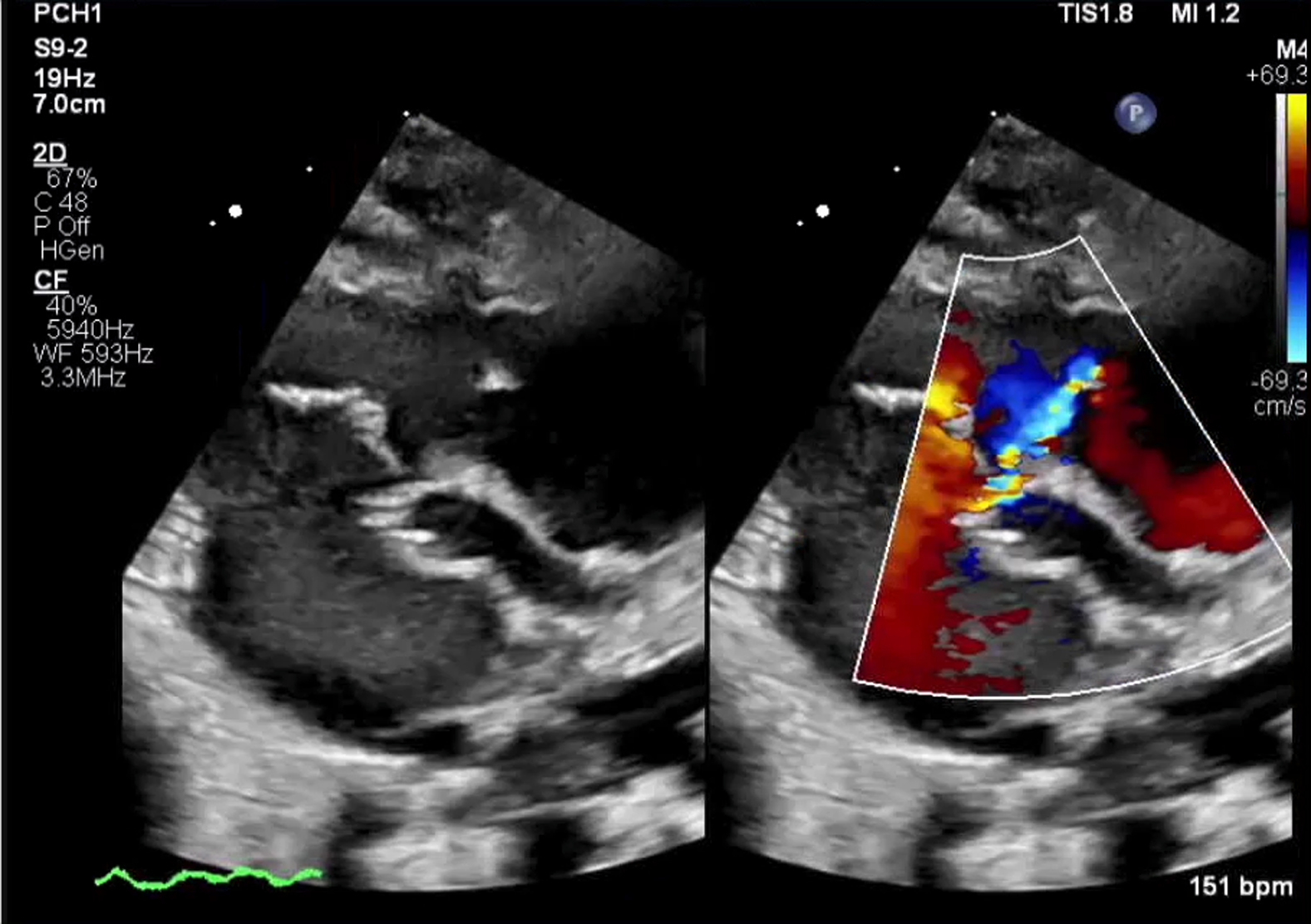

Supplement: Video 1 — Preoperative echocardiogram. Video available at: https://www.jtcvs.org/article/S2666-2507(26)00005-2/fulltext. [file fx2.jpg]

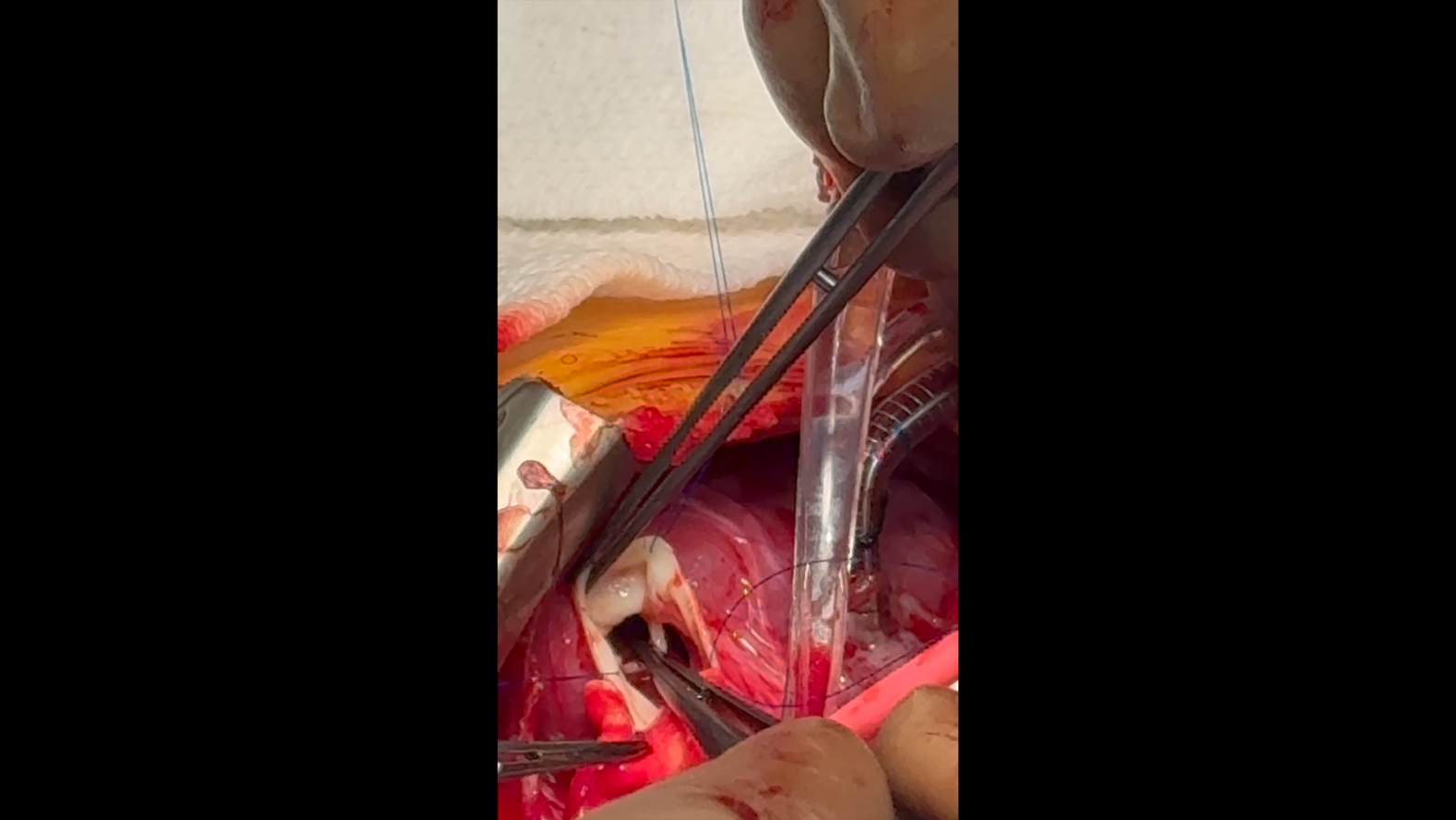

Supplement: Video 2 — Pulmonic valve cusp avulsion. Video available at: https://www.jtcvs.org/article/S2666-2507(26)00005-2/fulltext. [file fx3.jpg]

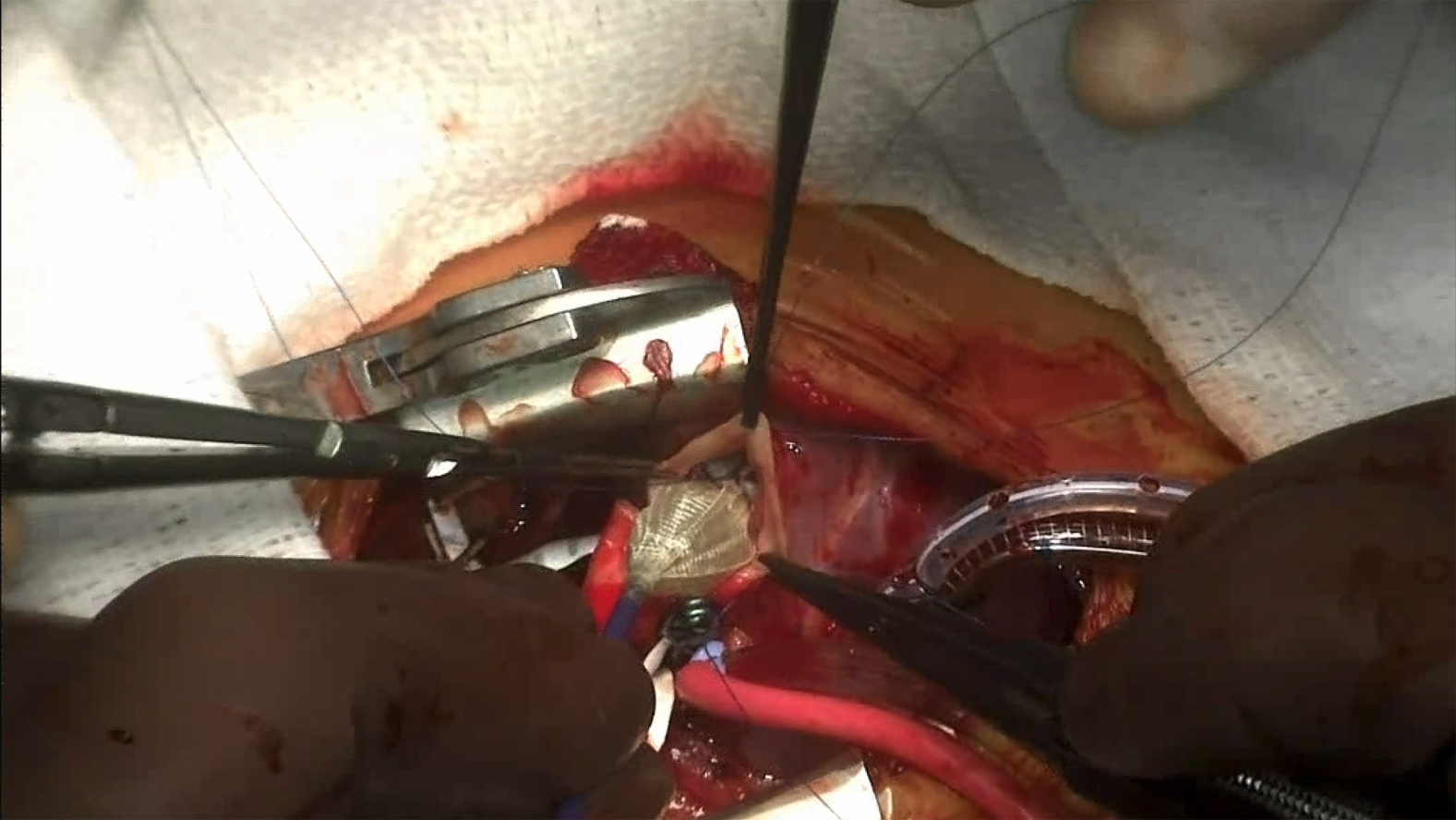

Supplement: Video 3 — Implantation technique of transcatheter valve into affected valve. Video available at: https://www.jtcvs.org/article/S2666-2507(26)00005-2/fulltext. [file fx4.jpg]
